# COVID-19 Vaccines and Myopericarditis: A Nuanced Story

**DOI:** 10.3390/jpm12081242

**Published:** 2022-07-29

**Authors:** Mark Jacobs, Andreas P. Kalogeropoulos

**Affiliations:** Division of Cardiology, Department of Medicine, Stony Brook University Medical Center, Health Sciences Center, T-16-080, 101 Nicolls Road, Stony Brook, NY 11794-8167, USA; mark.jacobs@stonybrookmedicine.edu

Vaccination has been a life-saving public health tool in the COVID-19 pandemic, with an estimated 19.8 million lives saved [1], as well as a safe one, with an estimated serious adverse event (SAE) rate of less than 0.1% [2]. An early concern for these vaccines has been the potential for myopericarditis. Posited mechanisms include mRNA immune reactivity, spike protein-myocardial protein cross-reactivity, and testosterone-mediated upregulation of immune response [3]. Nevertheless, large national databases have shown that the incidence of COVID-19 vaccine-associated myopericarditis has been rare [4].

In their work “Myocarditis and Pericarditis following COVID-19 Vaccination: Inequalities in Age and Vaccine Types” [5], Li and colleagues report on the demographic distribution of myopericarditis after COVID-19 vaccination with both mRNA and viral vector vaccines by combining data from the Vaccine Adverse Event Reporting System (VAERS) and the Centers for Disease Control and Prevention (CDC) COVID Data Tracker. This study was performed using reports of myopericarditis in the VAERS system between 11 December 2020 and 13 August 2021, creating an incidence using from the number of reported doses given from the CDC COVID Data Tracker. These results were then compared to all other vaccinations in the VAERS system. While the incidence of myopericarditis from COVID vaccination was overall rare (5.98 cases per million doses, 95% CI = 5.73–6.24), the incidence in the 12–17-year-old cohort was significantly higher (20.94 cases per million doses, 95% CI = 19.01–23.01). The incidence of myopericarditis was slightly higher among mRNA vaccine recipients compared to viral vector vaccine recipients (5.98 cases per million doses, 95% CI = 5.73–6.25 vs. 5.64 cases per million doses, 95% CI = 4.46–7.04), with Pfizer vaccine being associated with a higher incidence than Moderna vaccine (6.70 cases per million doses, 95% CI = 6.34–7.06 vs. 4.98 cases per million doses, 95% CI = 4.62–5.36). Of note, there was a higher incidence with the 2nd dose in both mRNA vaccines (Pfizer: 3.01 vs. 8.26 cases per million doses, Moderna: 3.09 vs. 5.13 cases per million doses). These findings merit further discussion below.

The authors are to be applauded for combining data from VAERS and CDC COVID Data Tracker, as this strengthens the validity of the denominator. However, it should also be noted that VAERS, which is a self-reporting system, has also a strong potential for self-selection [6]. The study used VAERS to provide a numerator for the number of events that occurred. VAERS is a portal designed as an early warning system for possible adverse side effects from vaccination as a means of monitoring safety. Prior studies have used VAERS as a numerator for the estimation of rare vaccine side effects such as anaphylaxis and Guillain-Barre [7]. This provides some reassurance about the validity of VAERS as a numerator in this case, as myopericarditis is a rare adverse event. Additionally, the incidence of myopericarditis in the study of Li et al. the has been estimated per dose, using the number of total dispensed doses in the US from the CDC COVID Data Tracker. Results are similar to other studies conducted using VAERS and the CDC COVID Data tracker, showing internal consistency [8].

Myopericarditis after vaccination is not a new finding with the COVID-19 or mRNA-based vaccines, as this complication has been seen in other vaccinations as well. Side effects such as chest pain and myopericarditis in both healthy individuals and those receiving treatments such as immunotherapy has been previously investigated for vaccinations such as influenza and smallpox [9,10]. A previous study of military age males aged 21–33 years of age revealed an incidence of 7.8 per 100,000 vaccinations, slightly higher than the similar age range of 18–64 in this study (5.9 cases per 100,000). Some prior studies of other vaccines have also used VAERS as an information source, which may mean that the under- or over-estimation of reports would be similar in either case [10]. However, prior prospective studies have shown that myopericarditis is likely underestimated in passive surveillance of adverse events after smallpox and influenza vaccination [9]. The rates of subclinical elevation in troponins in these studies suggest that myopericarditis with traditional vaccinations may be more common than previously noted.

Interestingly, there is a strong signal that myopericarditis after COVID-19 vaccines affects mostly young, male patients. Young males are particularly susceptible to developing myopericarditis [11]. Baseline incidence of myopericarditis, i.e., unrelated to vaccination, was found to be 2.18 per 100,000 (95% CI 1.90–2.34) in a study of military males ages 20–34 [10]. Prior investigations of sex-related differences in the development of this syndrome in the setting of acute viral illness has shown that testosterone has a pro-inflammatory effect, while estrogen may have a protective, anti-inflammatory effect [11,12,13]. These hormone-related effects may be responsible for the incidence differential across age and gender seen in the study of Li et al. [5].

Lower incidence of post-vaccination myopericarditis with viral vector COVID-19 vaccines has been also reported in a study from the United Kingdom [14]. That early study estimated an extra 2 (95% CI 0, 3), 1 (95% CI 0, 2) and 6 (95% CI 2, 8) myocarditis events per 1 million people vaccinated with ChAdOx1 (Oxford-AstraZeneca), BNT162b2 (Pfizer–BioNTech), and mRNA-1273 (Moderna), respectively, in the 28 days following a first dose and an extra 10 (95% CI 7, 11) myocarditis events per 1 million vaccinated in the 28 days after a second dose of mRNA-1273. Of note, a striking difference from Li et al. is that the estimated incidence of myopericarditis was considerably different between the two available mRNA vaccines [5].

This study observed an increased incidence of myocarditis after the second dose of mRNA vaccine than in the first, and in fact a lower incidence of myocarditis in the first dose of both mRNA vaccines compared to the single dose of the viral vector vaccine. Regulatory T-cells have been shown to be important in the development of myocarditis [15]. CD4+ T-cells have been shown to have increased differentiation with multiple exposures of antigen [16]. Repeated exposure to antigen may cause T-cell differentiation, autoimmunity, and the myopericarditis in these patients. Investigation of the incidence of myocarditis in those with viral vector vaccine boosters may help determine if the higher incidence in the second dose of mRNA vaccines is inherent to the vaccine modality vs. repeated exposure.

On the positive side, outcomes after COVID-19 vaccine-associated myopericarditis are favorable. In a large healthcare organization database from Israel, among 2.5 million vaccinees who received the Pfizer-BioNTech mRNA vaccine, there were 54 cases that met the criteria for myocarditis [4]. Among these cases, 3 out of 4 were mild and only 1 was associated with cardiogenic shock [4]. Left ventricular dysfunction was uncommon and resolved in half the cases in subsequent follow up imaging. In another report from Israel with the same vaccine, the presentation was judged to be mild in 95% of definite or probable cases and only 1 fulminant case was fatal [17]. Similarly low rates of adverse outcomes after COVID-19 vaccine-associated myopericarditis have been reported from other systems, including the United States [18].

In conclusion, most surveillance studies use data from self-reporting or secondhand reporting systems, e.g., VAERS, which does not have a standardized method of reporting adverse events. Denominators are usually approximated by using the overall number of doses delivered, as in the study of Li et al. However, there have been retrospective analyses from large healthcare databases that support similar generalized findings of myopericarditis occurring more likely in the age groups of younger males. Additionally, the findings of myopericarditis occurring more frequently after the second dose of the vaccine may be cause by repeated exposure in a short amount of time, whereas viral vector were only used once. It would be reasonable to consider viral vector vaccines in patients with prior episodes of myopericarditis and in young, male patients, as well as repeat analysis after booster doses of the viral vector vaccines. These types of studies may be useful in the future for studying the safety of new types of treatments for old or emerging diseases in an accelerated fashion.
